# Institutional Notes and News

**Published:** 1910-06-11

**Authors:** 


### Institutional Notes and News.

**The Queen Mother and Incurables.**

The Queen Mother has sent to the British Home and Hospital for Incurables, Streatham, the following letter, which was read after the meeting held in the Mansion House, presided over by the Lord Mayor "to inaugurate the jubilee" of the hospital: "Buckingham Palace, May 27, 1910.—Gentlemen, I have had the honour of submitting your letter to the Queen Alexandra with reference to the proposal of building a new wing to the British Home and Hospital for Incurables, at Streatham, in celebration of the Charity's Jubilee in 1911, and asking that the new wing may receive the name of Alexandra. I am now commanded by her Majesty to inform you that it will give her great pleasure to give permission for her name to be used.—I have the honour to be, gentlemen, your obedient servant, Sidney Greville." Mr. E. Penman, the Secretary, remarked at the meeting that the new Queen Alexandra wing would cost £30,000, though we understand that this sum is also to include a fund out of which outdoor pensions will be paid to deserving cases, whereby these may gain the little comforts which are valuable to invalids. The Bishop of Southwark moved a resolution to the effect that the meeting would use every effort to raise the money, and suggested that the Mayors should be invited to help by holding meetings in the towns they represented. The new wing will consist of four stories, the ground floor being given up to day-rooms and offices, and the remainder will accommodate forty more patients. The buildings, of which Mr. Edwin T. Hall, of Bedford Square, is the architect, are to be faced with red brick, a tile roof, and stone dressings.

**Colchester Hospital.**

An extraordinary meeting of the Essex County Hospital Committee was called recently, with Colonel Tyssen S. Holroyd in the chair, to consider the following motion which Colonel J. R. Chancellor introduced: "That in view of the serious deficit of £4,000 in the [Colchester] Hospital accounts, necessitating the sale of capital, it is advisable to recommend the Governors to reduce the expenditure by closing half the number of beds in all the wards, as there seems no prospect of future income balancing future expenditure on the present scale." In moving the resolution Colonel Chancellor pointed out that the last financial year ended with a debt of £4,066, of which £1,733 was the debt on that year. The proposed sale of their farms, if they realised their value, would more than wipe out last year's accumulations, but in about three years' time they would be in the same plight again. The present proposals of realising capital were like a company paying dividends from a similar source. We are glad to say that the following amendment was carried by a large majority: "That before any active steps are taken for closing any beds of the Hospital, or in any way curtailing the work of the out-patients' department, the matter should be referred to the Finance Committee for their consideration, to see what effect such a drastic measure would have on the finance of the Hospital." Mr. Ernest Sheldrake, the mover, who, we understand, has been appointed only recently to the Hospital Committee, said that he was strongly opposed to any curtailment of the benefits of the charity, either by closing beds or shutting up the out-patients' department. It is only fair to add that Mr. W. W. Hewitt, the hospital President, the Rev. E. Spurrier, Vice-Chairman of the Hospital Committee, and the Mayor of Colchester, Mr. E. Alec Blaxhill, all opposed the motion or supported the amendment, by letter, speeches, or votes.

**London Mental Hospital at Dartford.**

The City of London Mental Hospital at Dartford was fortunate in securing the Lord Mayor and the Lady Mayoress to open the new nurses' home, which has been built at a cost of nearly £6,000. Sir George Truscott (chairman of the Visiting Committee) remarked at the luncheon that in the last twelve years three Lord Mayors had served upon the Visiting Committee, and that each of them had been called upon to perform some ceremony at the asylum. Sir John Knill, in opening formally the new building, pointed out how important a relation nursing homes bear to hospitals, an importance which has been recognised only of late years. The nursing staff of the asylum, indeed, deserves to be housed properly, for it has a fine record, many of its nurses, in Sir George Truscott's words, having taken permanent posts in mental cases near and far. The asylum, which has accommodation for 600 patients, devotes half its space at present to private patients, there being some 300 of these in residence at the present time.

**Hitchin Hospital.**

The Hitchin Hospital has had a satisfactory year, in which much useful work has been done, and at the end of which is to be found a small balance at the bank. The total number of ward cases during the year has been 269, while 1,447 persons have attended at the out-patient department. Mr. E. J. Blain has been kept busy in the dental department of the hospital, and a well-deserved vote of thanks was given to him at the recent annual meeting. Certain additions were made during the year to the buildings, notably open-air shelters and further room for isolation cases, the cost of which was borne generously by Mr. T. Fenwick Harrison, who provided the £400 necessary to pay for them. The hospital's expenditure amounted to £1,374, and its total income £1,398, leaving a satisfactory balance-sheet. The hon. secretary, Mr. Lawson Thompson, was complimented on his work and constant interest in the institution. A committee is to be appointed to report upon the future policy of the hospital in the appointment of medical officers, and in their tenure of office. The Rev. Canon H. E. Jones must have been pleased to preside over an annual meeting which showed such a satisfactory state of affairs, and to head a secretary and staff of such willing workers.

**Holt Children's Sanatorium.**

Everyone will applaud the promise made by the King's Fund (referred to in our issue of December 18, 1909, p. 341) to head the subscription list for the Children's Sanatorium, Holt, with £500, since 70 out of the 105 children received so far have come from the Metropolis. The effects of the open-air life, which includes, logically enough, meals out of doors as often as our captious climate allows, have worked wonders, and we learn that, of twenty-four children who left the Sanatorium one or two years ago, fourteen are reported as maintaining their progress. So small, however, is the accommodation at present that though 107 applications were made in the second half of 1909, only eighteen of these could be granted admission. It is this demand for extended quarters and Dr. Gillam's report on the good effects which have crowned the Sanatorium's efforts which have led to the issue of an appeal for £6,000, so that the children may be housed permanently. We gather, however, that the word "housing" must be interpreted in the liberal sense which the open-air life implies, and there will be little reason to grumble if the buildings put up are more remarkable for size than solidity —solidity in the case of sanatoria and in the case of hospitals built on the pavilion system as they are to-day not being the object aimed at. We shall look forward to Dr. Gillam's reports in the future.

**London Hospital.**

The business before the recent quarterly Court of the London Hospital was on the whole satisfactory enough, as it included the record of a series of gifts of real value to the institution. First, Mr. Frederick Tendron's bequest of one-third of the residue of his estate, which is hoped to realise at least £8,000. Then a benefactor who conceals his name has given £1,000 to improve Tredegar House, which we have no doubt will be much appreciated by the pupil probationers, who, as everyone knows, spend the first period of their training there, before they face the wards. Finally, Princess Hatzfeldt has followed up her valuable present of a suite of baths for rheumatic patients with a promise of their extension. During the quarter, let us add, 22,299 persons had attended the out-patient department, and on the day the Court sat there were 803 patients in the wards. So the constructive work in the treatment of disease and in the improvement of the institution continues to thrive. But the workers themselves are hardly so immortal as the work, and we find Mr. Henry Fenwick, after 20 years' service as full surgeon, being appointed as consulting surgeon to the hospital. The London may be congratulated on retaining him and on having to record another honourable career successfully prolonged to embrace the chief honour it can give to its surgical staff.

**Adelaide Hospital, Dublin.**

Lord Langford, K.C.V.O., presided at the fifty-second annual meeting of the Adelaide Hospital, Dublin. Here is a hospital with a genuine balance at the bank on the right side of £310. In 1908 this balance was even larger, but last year there were calls upon the extraordinary expenditure account which make this reduction seem a matter of business rather than a matter of extravagance. The number of admissions in the past year was 1,372, and the "Marie Duckett" wing was opened and ready for them in the course of it. This wing meets the medical needs of the hospital, and has proved already that the £1,250 which it cost was well spent. The hospital's income amounted to £10,529 and the expenditure to £10,219. The rebuilding of the houses in Whitefriar Street and the purchase of the head rent of 28 Peter Street have enabled the committee to remark that, "out of current income in the past year, £1,900 has virtually been invested with profitable interest for the benefit of the hospital, leaving a surplus balance to credit and all necessities supplied. The committee is most thankful that it can show so satisfactory a balance-sheet, and sincerely thanks all those who have so liberally contributed to this result." The Fetherstonhaugh Convalescent Home, Rathfarnham, Co. Dublin, opened in 1894 by the late Lord Plunkett, is of incalculable value to the patients of the Adelaide Hospital. If, however, the public shows no greater signs of realising this than it has done lately, the Home's small endowment funds will have to be aided by drafts from the hospital's income, which would be disastrous for both institutions.

**Cumberland Infirmary.**

The Augmentation of Income Committee of the Cumberland Infirmary for the first quarter of the present year has now submitted its report, which has to lament the loss of old subscribers by death to an extent that is not covered wholly by the new subscribers who have been brought in. During the quarter the provision bill has been heavier owing to the increase of nearly eight per day in the number of patients. The daily average number of patients during the quarter has been ninety-four. The income over the same period is to all intents and purposes the same as it was during this part of 1909. Dr. Maclaren, in supporting the adoption of the quarterly statement, remarked that the necessary increase in income must very largely come from those who were receiving the benefits of the hospital. "We do not see our way to get more subscriptions from the rich, but we think that the working man should see that it was his interest very largely to increase his subscriptions." If the working man would make it his work to help the management rather than to criticise the management, Dr. Maclaren thought the hospital would be put upon a better foundation. The Building Fund has progressed so well that the children's ward will be completed. The amount now stands at over £26,000.

**Ashby Cottage Hospital.**

Mr. W. Dalglish-Bellasis, J.P., presided over the recent annual meeting of the Ashby-de-la-Zouch and District Cottage Hospital. He remarked that perhaps the best proof of the institution's usefulness was to be found in the fact that while three or four years ago minor operations only were performed, to-day dangerous operations are undertaken continuously with success. During the year 93 patients were treated. The hospital at present is supported by a mere handful of subscribers, the generosity of 65 persons supplying half the sum subscribed. There is a satisfactory surplus of £57 in hand, the income having been £441, and the expenditure £406, the last year, too, having begun with a balance. Subscribers of half a guinea qualify, under an altered rule, as governors, and by the Rev. W. Fowler's suggestion the annual meeting will be thrown open in future to all subscribers. Hitherto no genuine interest has been taken in it, chiefly, perhaps, because no one who gave less than one guinea was allowed to come.

**General Hospital, Tottenham.**

Only a few subscribers attended at the recent annual meeting of the Prince of Wales' General Hospital, Tottenham, but those who did attend listened to an account of useful work done in the course of the year. The enlarged wards were occupied fully for nearly all the year, and the daily average number of in-patients was 105, while 30,511 persons attended the out-patient department. The total income amounted to £10,133, and the total expenditure was £8,846, leaving a very satisfactory balance of £1,287. Unfortunately, however, an accumulated debt of £8,209 prevented the hospital retaining this advantage free of charge. The King's Fund, it should be remarked, gave the splendid sum of £4,000, and the Hospital Sunday and Saturday Funds gave over £1,000 between them. The hospital, like many others, complains of a scarcity of legacies, though the district of Tottenham is one surely which appeals to the generosity of any man who has taken the smallest trouble to discriminate between the different districts of London and the suburbs.

